# Custom-made foot orthoses: an analysis of prescription characteristics from an Australian commercial orthotic laboratory

**DOI:** 10.1186/s13047-017-0204-7

**Published:** 2017-06-07

**Authors:** Hylton B. Menz, Jamie J. Allan, Daniel R. Bonanno, Karl B. Landorf, George S. Murley

**Affiliations:** Discipline of Podiatry and La Trobe Sport and Exercise Medicine Research Centre, School of Allied Health, College of Science, Health and Engineering, La Trobe University, Melbourne, VIC Australia

**Keywords:** Orthotic devices, Foot, Prescriptions

## Abstract

**Background:**

Foot orthoses are widely used in the prevention and treatment of foot disorders. The aim of this study was to describe characteristics of custom-made foot orthosis prescriptions from a Australian podiatric orthotic laboratory.

**Methods:**

One thousand consecutive foot orthosis prescription forms were obtained from a commercial prescription foot orthosis laboratory located in Melbourne, Victoria, Australia (Footwork Podiatric Laboratory). Each item from the prescription form was documented in relation to orthosis type, cast correction, arch fill technique, cast modifications, shell material, shell modifications and cover material. Cluster analysis and discriminant function analysis were applied to identify patterns in the prescription data.

**Results:**

Prescriptions were obtained from 178 clinical practices across Australia and Hong Kong, with patients ranging in age from 5 to 92 years. Three broad categories (‘clusters’) were observed that were indicative of increasing ‘control’ of rearfoot pronation. A combination of five variables (rearfoot cast correction, cover shape, orthosis type, forefoot cast correction and plantar fascial accommodation) was able to identify these clusters with an accuracy of 70%. Significant differences between clusters were observed in relation to age and sex of the patient and the geographic location of the prescribing clinician.

**Conclusion:**

Foot orthosis prescriptions are complex, but can be broadly classified into three categories. Selection of these prescription subtypes appears to be influenced by both patient factors (age and sex) and clinician factors (clinic location).

**Electronic supplementary material:**

The online version of this article (doi:10.1186/s13047-017-0204-7) contains supplementary material, which is available to authorized users.

## Background

Foot orthoses are widely used in the prevention and treatment of foot and lower limb disorders [1, 2]. Globally, it has been estimated that the foot orthotic industry generated revenues of US$2.6 billion in 2015, with projections estimated at US$3.8 billion by 2021 due to population ageing and the increasing prevalence of chronic diseases such as diabetes [3]. Foot orthoses can be broadly categorised as *prefabricated* devices, which have a generic contour and can be purchased over the counter from health professionals, pharmacies or shoe stores, and *custom-made* devices, which are manufactured from a cast, impression or scan of an individual’s foot and are most commonly manufactured by commercial laboratories according to specifications requested by a podiatrist. However, this distinction is by no means absolute, as many prefabricated devices can be customised, and several custom orthotic laboratories offer ‘pre-cast’ orthoses with a limited selection of shell modifications.

The custom-made approach to orthotic therapy is based on the premise that by manufacturing foot orthoses with patient-specific design features, selected aspects of foot function can be modified in a therapeutically beneficial manner [4]. To facilitate this process, custom foot orthotic laboratories provide clinicians with a wide array of options to select from when prescribing orthoses, including different shell materials, degrees of cast correction, shell modifications such as cut-outs, skives, grooves and apertures, and a range of covering materials. However, there is only limited evidence that orthotic prescription variables alter foot function in a predictable, dose-response manner [5–7], and there is currently no broad consensus as to how foot orthoses should be prescribed [1, 8]. Consequently, orthotic prescriptions may vary considerably between clinicians.

Despite this, few studies have examined orthotic prescription approaches. In 2001, Landorf et al. [9] administered a questionnaire regarding orthosis prescription habits to 617 podiatrists in Australia and New Zealand. The majority of respondents (72%) prescribed custom-made orthoses most of the time, and the ‘typical’ prescription was a modified Root style orthosis, balanced to the neutral calcaneal stance position, with the shell made from polypropylene and an ethyl vinyl acetate (EVA) rearfoot post. More recently, Banwell et al. [10] conducted a clinical record audit of custom-made orthotic prescriptions for 42 patients with flexible pes planus in an Australian university podiatry clinic, and reported that 64% received orthoses posted to vertical, 36% were inverted, and 19% had a medial heel skive modification. Further examination of patient records indicated that patients who were prescribed a medial heel skive had a more everted resting calcaneal stance position, and those who were prescribed an inverted orthosis had a greater range of subtalar joint motion, suggesting that the selection of these prescription options was guided by individual patient requirements. Another recent, qualitative study by Williams et al. [8] concluded that orthotic prescription approaches were highly variable between clinicians, with ‘trial and error’ and previous experience playing a larger role in informing clinical practice than research evidence.

Although these studies provide useful insights into prescription patterns, they are limited by time period (the Landorf et al. survey being conducted over 15 years ago) and scope (the Banwell et al. study being limited to one university clinic and the Williams et al. study only including 16 participants). Therefore, to provide a more contemporary and representative picture of prescription patterns, we analysed 1000 prescriptions from a large commercial orthotic laboratory. In doing so, our objectives were to: (i) describe the frequency of individual prescription variables, (ii) determine whether prescriptions could be broadly classified into subgroups, and (iii) explore whether the identified subgroups differed according to patient-specific and clinician-specific factors.

## Methods

**Fig. 1:**
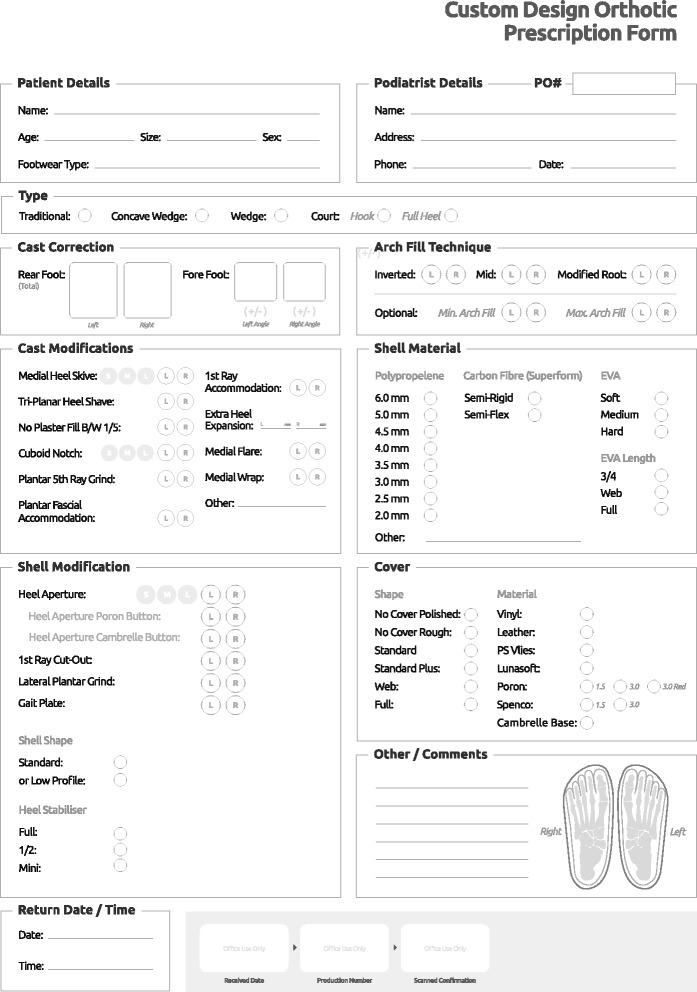
The Footwork Podiatric Laboratory prescription form. Detailed descriptions of each item can be accessed at the Footwork Podiatric Laboratory YouTube channel [11]

Ethical approval was provided by the La Trobe University College of Science, Health and Engineering Human Ethics Sub-Committee (Reference: S15/83). One thousand consecutive foot orthosis prescription forms received in 2015 were provided by a commercial prescription foot orthosis laboratory located in Melbourne, Victoria, Australia (Footwork Podiatric Laboratory). All forms were first de-identified in relation to both prescribing clinician and patient details. Each item from the prescription form was documented for both right and left feet in relation to: (i) orthosis type, (ii) cast correction, (iii) arch fill technique, (iv) cast modifications, (v) shell material, (vi) shell modifications and (vii) cover shape/material. The age and sex of the patient and location of the podiatrist were also documented. The prescription form is shown in Fig. 1, and detailed explanations of each prescription variable can be viewed at the Footwork Podiatric Laboratory YouTube channel [11].

### Statistical analysis

Statistical analysis was undertaken using SPSS Version 22.0 (IBM, Armonk, NY, USA). The analysis was undertaken in five stages. First, to test for symmetry, associations between right and left foot orthosis prescription variables were analysed using Spearman’s rho correlation coefficient. Second, simple descriptive analysis was conducted to analyse the frequencies of each prescription feature. Third, to determine whether the orthosis prescriptions could be collapsed into groups, a cluster analysis was undertaken. This involved entering each of the prescription variables into the *k*-means cluster function in SPSS and pre-specifying the number of output clusters (for this analysis, we selected three). Fourth, to determine which prescription variables best discriminated between the three identified clusters, a discriminant function analysis was performed. This involved entering each of the prescription variables that were significantly different between the clusters into a step-wise discriminant function model, and the relative importance of each variable in discriminating between responders and non-responders was determined using standardised canonical discriminant function coefficients. After deriving the discriminant function, the accuracy of the model in identifying clusters was expressed as a percentage. Finally, to characterise the three clusters in relation to differences in individual prescription variables, patient and clinician characteristics, chi-square (for nominal or ordinal variables) and one-way analyses of variance (for continuous variables) were used.

## Results

### Prescription dataset

One thousand prescriptions were extracted and analysed from 178 different clinical practices. Of these, 983 (98%) had the location of the practice documented. Most prescriptions from Australia were from the state of Victoria (*n* = 726, 73.9%), followed by New South Wales (*n* = 106, 10.8%), the Australian Capital Territory (*n* = 49, 5.0%), Western Australia (*n* = 36, 3.7%), Queensland (*n* = 28, 2.8%), Tasmania (*n* = 27, 2.7%) and South Australia (*n* = 1, 0.1%). There were also a small number of prescriptions from Hong Kong (*n* = 10, 1.0%). The sex and age of the patient were documented in 984 (98.4%) and 699 (69.9%) prescriptions, respectively. There were 583 prescriptions for female and 401 for male patients, and patient age ranged from 5 to 92 years (mean 41.4 years, SD 21.4).

### Symmetry

Prescription variables were highly symmetrical, with Spearman’s rho correlation coefficients ranging from 0.873 to 0.990 (*p* < 0.01 for all correlations). Therefore, all subsequent analyses were performed using the right foot only [12].

### Frequencies of individual prescription variables

The frequencies of each prescription variable are summarised below. For variables with missing data, the percentage reported is the valid percentage (i.e. percentage of available data, excluding missing cases).

#### Orthosis type

The most commonly prescribed orthosis type was traditional (*n* = 493, 51.0%), followed by concave wedge (*n* = 420, 42.0%), court with full heel (*n* = 28, 2.9%), court with hook heel (*n* = 16, 1.7%) and wedge (*n* = 10, 1.0%).

#### Cast correction

Cast correction for the rearfoot ranged from 11 degrees valgus to 35 degrees varus (mean 6.3, SD 5.5), and for the forefoot, from 10 degrees valgus to 30 degrees varus (mean − 0.1, SD 1.4). The most frequently selected rearfoot cast correction was 0 degrees (*n* = 161, 16.1%) and the vast majority of cast corrections for the forefoot were 0 degrees (*n* = 895, 89.5%).

#### Arch fill technique

The most commonly prescribed arch fill technique was mid (*n* = 472, 49.0%), followed by mid-modified Root (*n* = 168, 17.4%), inverted-mid (*n* = 152, 15.8%), modified Root (*n* = 101, 10.5%), inverted (*n* = 55, 5.7%), inverted-modified Root (*n* = 9, 0.9%), inverted-mid-modified Root (*n* = 5, 0.5%) and other (*n* = 2, 0.2%). The additional option of minimum or maximum arch fill was selected for 180 (18.1%) and 43 (4.3%) prescriptions, respectively.

#### Cast modifications

Cast modifications were requested in 789 (78.9%) prescriptions. The most commonly prescribed cast modification was plantar fascial accommodation (*n* = 351, 35.1%), followed by cuboid notch (*n* = 334, 33.4%), 1st ray accommodation (*n* = 211, 21.1%), plantar 5th ray grind (*n* = 204, 20.4%), medial flare (*n* = 142, 14.2%), no plaster fill between metatarsal heads 1 to 5 (*n* = 102, 10.2%), medial heel skive (*n* = 95, 9.5%), heel expansion (*n* = 45, 4.5%), triplanar heel skive (*n* = 23, 2.3%), lateral heel skive (*n* = 14, 1.4%) and medial wrap (*n* = 15, 1.5%).

#### Shell material

The most commonly prescribed shell material was polypropylene (*n* = 913, 92.4%), followed by EVA (*n* = 41, 4.1%) and carbon fibre/TL-2100 (*n* = 4, 0.4%). The thickness of polypropylene ranged from 1 to 6 mm, with the most commonly prescribed thickness being 3 mm (*n* = 285, 31.2%).

#### Shell modifications

Shell modifications were requested in 824 (82.4%) prescriptions. The most commonly prescribed shell modification was a heel stabiliser (*n* = 739, 73.9%), which was commonly manufactured from polypropylene (*n* = 652, compared to *n* = 87 from EVA). The next most commonly prescribed shell modification was a low profile shell (*n* = 171, 17.1%), followed by a heel aperture (*n* = 91, 9.1%), 1st ray cut-out (*n* = 61, 6.1%), lateral plantar grind (*n* = 39, 3.9%) and gait plate (*n* = 11, 1.1%).

#### Cover shape and material

Covers were requested in 782 (93.9%) prescriptions. The most common cover length was full (*n* = 520, 66.5%), followed by standard (*n* = 105, 13.4%), web (*n* = 83, 10.6%) and standard plus (*n* = 74, 9.5%). There were 32 different cover material prescriptions. The most commonly requested cover material was PORON® 1.5 mm plus nora® Lunasoft (*n* = 180, 21.8%), followed by leather (*n* = 129, 15.7%) and nora® Lunasoft alone (*n* = 104, 12.6%).

### Cluster analysis

The 3 cluster model converged after 12 iterations. The number of prescriptions in each cluster was as follows: cluster 1 (*n* = 224), cluster 2 (*n* = 540) and cluster 3 (*n* = 236). Table 1 shows the prescription characteristics of the 3 clusters. Significant differences between the clusters were observed in relation to orthosis type, cast correction (rearfoot and forefoot), arch fill technique, medial heel skive, triplanar heel shave, no plaster fill between metatarsophalangeal joints 1 to 5, cuboid notch, plantar fascial accommodation, medial flare, lateral plantar grind, low profile shell, heel stabiliser and cover shape.

**Table 1:** Prescription characteristics of the three clusters

	Cluster 1 (*n* = 224)	Cluster 2 (*n* = 540)	Cluster 3 (*n* = 236)	*p*
Orthosis type				
Traditional^a^	119 (56.1)	291 (55.5)	83 (35.2)	<0.001
Concave wedge^a^	82 (38.7)	211 (40.3)	127 (55.0)	<0.001
Wedge	3 (1.4)	2 (0.4)	5 (2.2)	0.068
Court (hook)	3 (1.4)	12 (2.3)	1 (0.4)	0.174
Court (full)^a^	5 (2.4)	8 (1.5)	15 (6.5)	0.001
Cast correction in degrees – rearfoot^b^				
-11 to 2^a^	56 (25.0)	187 (34.6)	0 (0.0)	<0.001
3 to 5^a^	68 (30.4)	224 (41.5)	0 (0.0)	<0.001
6 to 9^a^	71 (31.7)	129 (23.9)	5 (2.1)	<0.001
10 to 35^a^	29 (12.9)	0 (0.0)	231 (97.9)	<0.001
Cast correction in degrees – forefoot^c^				
-10 to -1^a^	10 (4.5)	46 (8.5)	7 (3.0)	0.006
0^a^	194 (21.7)	478 (88.5)	223 (94.5)	0.012
1 to 30^a^	20 (8.9)	16 (3.0)	6 (2.5)	<0.001
Arch fill technique				
Modified Root^a^	31 (14.0)	65 (12.2)	5 (2.1)	<0.001
Mid^a^	109 (49.1)	334 (62.9)	29 (12.3)	<0.001
Mid-modified Root^a^	56 (25.2)	89 (16.8)	23 (9.7)	<0.001
Inverted^a^	2 (0.9)	3 (0.3)	50 (5.1)	<0.001
Inverted-mid^a^	12 (5.4)	24 (4.5)	116 (49.2)	<0.001
Inverted-modified Root^a^	1 (0.5)	0 (0.0)	8 (3.4)	<0.001
Inverted mid-modified Root^a^	1 (0.5)	0 (0.0)	4 (1.7)	0.011
Arch fill – optional				
Minimum	48 (21.6)	94 (17.5)	38 (16.1)	0.272
Standard	168 (75.7)	417 (77.8)	186 (78.8)	0.389
Maximum	6 (2.7)	25 (4.7)	12 (5.1)	0.710
Cast modifications				
Medial heel skive^a^	22 (9.8)	72 (13.3)	9 (3.8)	<0.001
Lateral heel skive^a^	4 (1.8)	8 (1.5)	2 (0.8)	0.674
Triplanar heel shave^a^	3 (1.3)	19 (3.5)	1 (0.4)	0.017
No plaster fill between 1-5^a^	32 (14.3)	60 (11.1)	10 (4.2)	<0.001
Cuboid notch^a^	66 (29.5)	174 (32.2)	94 (39.8)	0.043
Plantar 5th ray grind	34 (15.2)	114 (21.1)	56 (23.7)	0.063
Plantar fascial accommodation^a^	95 (42.4)	174 (32.2)	82 (34.7)	0.027
1st ray accommodation	39 (17.4)	119 (22.0)	53 (22.5)	0.305
Heel expansion	15 (6.7)	17 (3.1)	13 (5.5)	0.068
Medial flare^a^	33 (14.7)	50 (9.3)	59 (25.0)	<0.001
Medial wrap	6 (2.7)	6 (1.1)	3 (1.3)	0.254
Shell material				
Polypropylene	202 (92.2)	489 (91.6)	222 (94.9)	0.246
Carbon fibre / TL2100	1 (0.5)	3 (0.6)	0 (0.0)	0.525
EVA	16 (7.3)	43 (8.1)	12 (5.1)	0.355
Shell modifications				
Heel aperture	26 (11.6)	47 (8.7)	18 (7.6)	0.298
1st ray cut-out	14 (6.3)	35 (6.5)	12 (5.1)	0.752
Lateral plantar grind^a^	4 (1.8)	9 (1.7)	26 (11.0)	<0.001
Gait plate	3 (1.3)	5 (0.9)	3 (0.3)	0.847
Low profile shell^a^	31 (13.9)	80 (14.8)	60 (25.4)	0.002
Heel stabiliser^a^	167 (74.6)	372 (68.9)	194 (82.2)	<0.001
Cover shape				
No cover – polished^a^	1 (0.6)	20 (4.2)	4 (2.1)	0.041
No cover – rough	3 (1.8)	15 (3.2)	8 (4.2)	0.400
Standard^a^	3 (1.8)	75 (15.9)	27 (14.3)	<0.001
Standard plus^b^	5 (2.9)	53 (11.2)	16 (9.5)	0.005
Web	10 (5.8)	55 (11.6)	18 (9.5)	0.094
Full^b^	149 (87.1)	255 (53.9)	116 (61.4)	<0.001

^a^significant difference between clusters according to chi-square analysis

^b^variable divided into quartiles for analysis

^c^ variable divided into tertiles for analysis

Values are n (%)

Cluster 1 was characterised by a prescription more likely to be a traditional orthosis type, 6 to 9 degrees of varus rearfoot cast correction, 1 to 30 degrees of varus forefoot cast correction, modified Root or mid-modified Root arch fill technique, no plaster fill between 1st and 5th metatarsal heads, a plantar fascial accommodation, and a full length cover.

Cluster 2 was characterised by a prescription more likely to be a traditional orthosis type, 11 degrees valgus to 5 degrees varus rearfoot cast correction, 10 degrees valgus to 0 degrees of forefoot cast correction, incorporate a mid arch fill technique, a medial heel skive or triplanar heel shave, no cover-polished standard or standard plus cover.

**Fig. 2:**
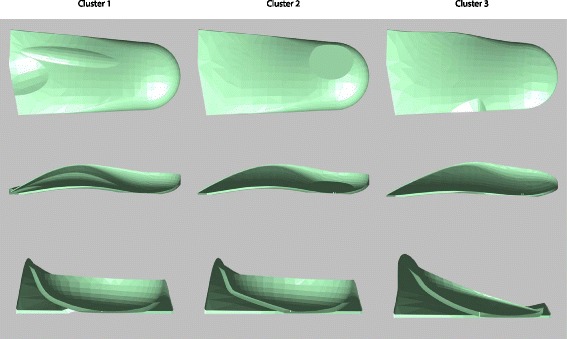
Three dimensional representations of typical orthoses for each of the identified clusters, generated from the same patient foot scan. Top: plantar view, middle: medial view, bottom: posterior view (medial side to left). NB: heel stabilisers have been removed to aid visualisation of the heel contour and non-automated finishing (e.g. rounding of anterior edge of orthosis) not included

Cluster 3 was characterised by a prescription more likely to be a concave wedge or full court orthosis type, 10 to 35 degrees of varus cast correction in the rearfoot, 0 degrees of cast correction in the forefoot, an inverted, inverted-mid, inverted-modified Root or inverted mid-modified Root arch fill technique, a cuboid notch, medial flare, lateral plantar grind, low profile shell and heel stabiliser. The characteristics of these clusters are summarised in Fig. 2, and the 3-dimensional stereolithography (STL) files can be downloaded for viewing (see Additional files 1, 2 and 3).

The step-wise discriminant function analysis was significant (λ = 0.77, χ^2^ = 863.8, *p* < 0.001) and the final model indicated that that 5 variables significantly discriminated between the 3 clusters: rearfoot cast correction (λ = 0.82), cover shape (0.37), orthosis type (0.36), forefoot cast correction (0.35) and plantar fascial accommodation (0.35). The combination of these five variables was able to identify cluster membership with an accuracy of 70%.

Table 2 shows the patient and practitioner characteristics of the 3 clusters. Cluster 1 prescriptions were more likely to have been requested from clinicians in New South Wales, the Australian Capital Territory and Hong Kong. Cluster 2 prescriptions were more likely to have been requested by clinicians in Western Australia and included a higher proportion of female patients. Cluster 3 prescriptions were more likely to have been requested by clinicians in Victoria, included a higher proportion of male patients, and were prescribed for significantly younger patients.

**Table 2:** Patient and practitioner characteristics of the three clusters

	Cluster 1 (*n* = 224)	Cluster 2 (*n* = 540)	Cluster 3 (*n* = 236)	*p*
Patient				
Sex^a^				
Male	96 (42.9)	195 (36.7)	110 (48.2)	0.009
Female	128 (57.1)	337 (63.3)	118 (51.8)	
Age – mean (SD) years^a^	44.4 (22.1)	44.0 (19.9)	33.3 (21.6)	<0.001
Practitioner				
Location				
Victoria^a^	130 (58.0)	416 (77.0)	180 (76.3)	<0.001
New South Wales^a^	31 (13.8)	45 (8.3)	30 (12.7)	0.041
ACT^a^	34 (15.2)	8 (1.5)	7 (3.0)	<0.001
Queensland	8 (3.6)	12 (2.2)	8 (3.4)	0.491
Western Australia^a^	3 (1.3)	30 (5.6)	3 (1.3)	<0.001
Tasmania	8 (3.6)	17 (3.2)	2 (0.9)	0.132
South Australia	1 (0.4)	0 (0.0)	0 (0.0)	0.182
Hong Kong^a^	8 (3.6)	2 (0.4)	0 (0.0)	<0.001

^a^significant difference between clusters according to chi-square analysis. ACT = Australian Capital Territory

Values are n (%) unless otherwise stated

## Discussion

The objective of this study was to describe patterns of custom-made foot orthosis prescriptions from a commercial Australian podiatric orthotic laboratory, in order to provide insights into contemporary clinical practice. Overall, we found that although a wide array of prescription variables are being utilised by clinicians, the basic design features of orthoses could be broadly classified into three subtypes (‘clusters’), primarily dictated by variation in rearfoot cast correction. We also found significant differences between these clusters in relation to the age and sex of the patient, and the geographic location of the prescribing clinician, indicating that both patient- and clinician-specific factors influence prescribing patterns.

When the frequency of individual prescription items are considered, our findings are similar to those of Landorf et al. [9], who found that the ‘typical’ orthosis prescription was a modified Root style functional foot orthosis, constructed from polypropylene, and balanced to the neutral or vertical calcaneal stance position with a heel stabiliser (also referred to as a rearfoot post). We also found polypropylene to be the most frequently prescribed material (92% of prescriptions), the most frequently selected rearfoot cast correction was 0 degrees (16% of prescriptions) and that the majority of prescriptions included a heel stabiliser (73.9%). The predominance of polypropylene observed is consistent with a recent industry analysis that suggested that polypropylene accounts for 43% of the entire foot orthotic market [3].

However, the statistical approach we employed in this study also allowed us to identify three broad ‘clusters’ of orthosis based on the inclusion of multiple prescription variables, thereby providing more detailed insights into prescription approaches. It is important to note that these clusters should not be considered to represent completely distinct, homogenous groups, as considerable variability is evident within each cluster. A more correct interpretation is that orthotic prescriptions within each cluster are more similar to each other than prescriptions in the other two clusters. The observation of clustering suggests that although thousands of prescription combinations could potentially be generated from an orthotic prescription form, it is likely that in practice far fewer combinations are actually utilised due to the interdependence of individual prescription variables.

The characteristics of the three identified clusters are summarised in Fig. 2. Based on the theorised biomechanical effects of these prescription variables, these clusters could be considered to represent orthosis designs which progressively increase supination moments (i.e. increased levels of pronation ‘control’). Cluster 1, characterised by a prescription more likely to incorporate a modified Root or mid-modified Root arch fill technique and a higher cast correction in the forefoot, could be considered to be similar to the foot orthosis described by Root [13]. Cluster 2 could be considered to provide greater rearfoot pronation ‘control’ than cluster 1 due to the combination of the mid arch fill technique (where the highest point of the orthosis is located at the talo-navicular joint) and a medial heel skive, an orthotic modification that aims to increase the supination moment acting across the subtalar joint axis [14]. Finally, cluster 3 features even greater rearfoot pronation control features than cluster 1 and 2, as it is characterised by a prescription more likely to incorporate an inverted arch fill technique and higher cast correction in the rearfoot, similar to the inverted device first described by Blake [15, 16].

The observation of these three broad ‘clusters’ of orthoses is consistent with the recent findings of Banwell et al. [17], who conducted a Delphi survey to establish the rationale underpinning orthotic prescription by Australian podiatrists for symptomatic flexible pes planus. Clinicians were asked to indicate what type of foot orthosis they would typically prescribe in the presence of clinical signs of pronated foot posture, classified as ‘moderate’ or ‘considerable’ rearfoot eversion, talonavicular bulging and lowered navicular position. The orthotic options were modified Root device posted to neutral, modified Root device posted to inverted, or Inverted device (i.e. Blake Inverted). For ‘moderate’ signs of pronated foot posture, clinicians were more likely to prescribe a modified Root orthosis (neutral or inverted), while for ‘considerable’ signs of pronated foot posture, clinicians were more likely to prescribe an Inverted orthosis. Furthermore, clinicians reached consensus that when increased control of foot pronation is required, the orthosis should be an Inverted device (i.e. incorporate an inverted correction) or include a medial heel skive (i.e. medial heel skive modification to the cast).

The discriminant function analysis indicated that the three clusters could be identified by a combination of 5 variables (rearfoot cast correction, cover shape, orthosis type, forefoot cast correction and plantar fascial accommodation), with an overall prediction accuracy of 70%. This suggests that although the clusters were initially derived from 48 different prescription variables, a significant amount of variance in prescriptions can be explained by a much smaller subset of variables, with rearfoot cast correction being the strongest predictor. The remaining variance can be explained by variables such as covering material and less frequently requested optional additions, such as heel lifts and deflective forefoot padding. While these variables would influence the function of the orthosis, they appear to make less of a contribution to identifying the three broad types of device.

When the three clusters were compared, we found significant differences in relation to clinic location and the age and sex of the patient. It is possible that clinic location may be a proxy indicator of educational background, in that clinicians in the same state or country may be more likely to have attended the same university and therefore adopted similar orthotic prescribing approaches compared to clinicians in other geographic locations. The associations with age and sex suggest that older patients and females may be less likely to be prescribed more ‘controlling’ orthoses. The association with age may reflect caution regarding the possible detrimental effects of highly controlling foot orthoses on skin integrity or balance in an older person [18, 19]. However, the association with sex is unclear, as although there are some morphological differences between the feet of men and women [20, 21], no sex-specific differences in foot posture have been reported [22, 23] and there is no evidence that responses to foot orthoses differ according to sex.

Our findings need to be interepreted in the context of several limitations. First, the prescription data were obtained from a single laboratory in the state of Victoria, Australia. Given that the definition and interpretation of individual prescription items may vary, our findings cannot necessarily be generalised to other laboratories, both within Australia and other countries. This is particularly the case for the ‘arch fill’ option, the terminology of which is unique to the laboratory we used. Second, the statistical analysis approach required that the number of clusters be pre-specified. We selected three clusters based on the premise that there would be three broad ‘types’ of orthosis: a modified Root-style device, and inverted-style device, and a ‘hybrid’ device somewhere in between. Our results confirmed this and revealed the three clusters are well delineated. However, several other cluster solutions could be derived from these data with different interpretations as to their meaning. Third, we have interpreted the three clusters using a rather simplistic paradigm of increasing ‘control’ of foot pronation. Although this is consistent with previous literature pertaining to the indications for prescribing orthotic modifications such as inverted orthoses and medial heel skives [10, 14, 15, 17], we acknowledge that this is not the only goal of orthotic therapy and that the clinical reasoning underpinning these prescriptions may be far more complex. Finally, as we did not have access to detailed clinical assessment data or patient-reported outcomes, we are unable to comment as to how each individual’s biomechanical profile influenced the prescription, nor whether these prescriptions were ‘correct’ or appropriate.

The findings of this study have implications for both clinical practice and research. For clinicians, these data provide a point of reference against which their individual orthotic prescription approach can be benchmarked. For researchers, these findings can be used to inform the selection of prescription parameters to ensure that the orthoses used in future clinical trials are reflective of contemporary clinical practice.

## Conclusion

This study has shown that although foot orthosis prescriptions are complex, they can be broadly classified into three subtypes, the selection of which is influenced by both patient- and clinician-specific factors. These findings provide useful insights into the clinical practice of orthotic therapy and may assist in the design of future clinical trials of foot orthoses.

## Additional files

Additional file 1: Stereolithography (STL) file of cluster 1 orthosis. To view the 3D model, download the file and drag and drop at http://www.viewstl.com/. (STL 823 kb)

Additional file 2: Stereolithography (STL) file of cluster 2 orthosis. To view the 3D model, download the file and drag and drop at http://www.viewstl.com/. (STL 634 kb)

Additional file 3: Stereolithography (STL) file of cluster 3 orthosis. To view the 3D model, download the file and drag and drop at http://www.viewstl.com/. (STL 611 kb)

## Abbreviations

ACT: Australian Capital Territory
EVA: Ethyl vinyl acetate
SD: Standard deviation
STL: Stereolithography
